# Net Efficacy Adjusted for Risk (NEAR): A Simple Procedure for Measuring Risk:Benefit Balance

**DOI:** 10.1371/journal.pone.0003580

**Published:** 2008-10-31

**Authors:** José N. Boada, Carlos Boada, Mar García-Sáiz, Marcelino García, Eduardo Fernández, Eugenio Gómez

**Affiliations:** Clinical Pharmacology Unit, Pharmacovigilance Centre, University Hospital of Canary Islands, University of La Laguna, La Laguna, Santa Cruz de Tenerife, Spain; Harvard School of Public Health, United States of America

## Abstract

**Background:**

Although several mathematical models have been proposed to assess the risk:benefit of drugs in one measure, their use in practice has been rather limited. Our objective was to design a simple, easily applicable model. In this respect, measuring the proportion of patients who respond favorably to treatment without being affected by adverse drug reactions (ADR) could be a suitable endpoint. However, remarkably few published clinical trials report the data required to calculate this proportion. As an approach to the problem, we calculated the expected proportion of this type of patients.

**Methodology/Principal Findings:**

Theoretically, responders without ADR may be obtained by multiplying the total number of responders by the total number of subjects that did not suffer ADR, and dividing the product by the total number of subjects studied. When two drugs are studied, the same calculation may be repeated for the second drug. Then, by constructing a 2×2 table with the expected frequencies of responders with and without ADR, and non-responders with and without ADR, the odds ratio and relative risk with their confidence intervals may be easily calculated and graphically represented on a logarithmic scale. Such measures represent “net efficacy adjusted for risk” (NEAR).

We assayed the model with results extracted from several published clinical trials or meta-analyses. On comparing our results with those originally reported by the authors, marked differences were found in some cases, with ADR arising as a relevant factor to balance the clinical benefit obtained. The particular features of the adverse reaction that must be weighed against benefit is discussed in the paper.

**Conclusion:**

NEAR representing overall risk-benefit may contribute to improving knowledge of drug clinical usefulness. As most published clinical trials tend to overestimate benefits and underestimate toxicity, our measure represents an effort to change this trend.

## Introduction

Although several mathematical models have been proposed to assess risk:benefit balance of drugs in one measure [1]–[4], their use in practice has been rather limited. Difficulties in computation as well as in interpreting results may have hampered a broader use of these formulations. Our objective was to design a simple, easily applicable model to overcome such obstacles. To this end, the following criteria were considered important: a) data for computation must be easily available; b) risk and benefit must be combined in a single measure, and c) this measure must be sufficiently clear to be used in clinical practice.

As to these criteria, data for computation may be extracted from well known sources, mainly from controlled randomized clinical trials (CRT) and their meta-analytical reviews; secondarily, results of observational prospective studies may also be used.

In order to combine efficacy and safety in one easily understandable measure, results obtained from CRT may be divided into two categories: a) patients responding to treatment without suffering adverse drug reactions (ADR); and b) the remaining patients, i.e. the sum of those responding to the drug but suffering ADR, plus those resistant to the treatment but not suffering ADR, plus those resistant to the treatment and simultaneously suffering ADR. It is clear that the proportion of patients who respond favorably to treatment without being affected by ADR is an easily understandable parameter representing the optimum effect of the drug. However, in practice, most published data sources fail to report this detail. In addition, slight ADR do not have the same weight as severe ADR. Consequently, both problems require discussion and a solution.

Leaving aside for a posterior analysis those aspects related to the severity of ADR, the number of patients responding to treatment without suffering ADR is unavailable in most published data sources, as mentioned above. Indeed, efficacy and safety data are normally reported separately, as if they had been obtained from different populations; moreover, the units of measurement frequently correspond to non-comparable scales of benefit and risk. Our approach to this problem was to use a statistical maneuver i.e. expected frequency calculation, which is a well known step in chi square calculation. In this manner, we were able to determine the theoretical proportion of patients favorably responding to the drug without suffering ADR. This measure may be useful, mainly when two drugs are compared.

As to the question of what type of ADR must be weighed against the benefit endpoint, the simplest answer is you can do what you like. This sounds a little unorthodox but it is the best answer in practical terms, as will be shown below. In fact there are no standardized and universal rating scales for categorizing the severity of ADR. Therefore, selecting an ADR to calculate risk:benefit balance is better done by taking into consideration each particular clinical problem. For instance, in selecting between two opioid analgesics for palliative care, perhaps the ADR that physicians most like to avoid is vomiting, which, although not a severe ADR, may hinder the therapeutic efficacy and decrease patient quality of life. Although detailed severity analysis may be interesting from an academic or a regulatory point of view, clinical condition is decisive at the time of selecting what type of drug therapy must be employed.

Once the above mentioned problems are solved, details about computation and its application to several practical cases are shown below.

## Methods

As already indicated, theoretically, results obtained in either CRT or observational studies can be separated into the categories shown in Table 1.

**Table 1:** 2×2 table combining efficacy and safety results of a theoretical CRT.

	Responders	Non-responders	
Without ADR	a	b	Total without ADR
Suffering ADR	c	d	Total suffering ADR
	Total responders	Total non-responders	Total studied

Whenever these data are originally reported, it is feasible to calculate the odds ratio (OR) or relative risk (RR); in these cases safety and efficacy are directly combined. But published studies practically never present these data, and, consequently, such tables can not be constructed.

As explained before, a statistical manner of addressing the problem consists in replacing the observed frequencies by their expected ones. The procedure is feasible whenever the total number of patients who responded to treatment favorably as well as the total number of patients who experienced ADR are reported; happily, authors do usually report these data. So, the expected frequency for cell “a” in Table 1 is obtained by multiplying the total number of responders by the total number of subjects that did not suffer ADR, and dividing the product by the total number of subjects studied. Similar calculations may be performed to obtain the expected frequencies for the remaining cells.

When two drugs are involved, which is the most frequent case in practice, all the above calculations may be repeated for the second drug. Once the expected frequencies for the two treatments are obtained the data may be then organized in the manner indicated in Table 2.

**Table 2:** Table combining efficacy and safety results of a theoretical CRT when two drugs have been studied.

	Responders without ADR	Responders with ADR	Non-responders without ADR	Non-responders with ADR	Totals
Treatment A	a_1_	c_1_	b_1_	d_1_	n_1_
Treatment B	a_2_	c_2_	b_2_	d_2_	n_2_

Thus, the optimum effect corresponds to the cells in the first column, i.e., a_1_ and a_2_. Now, to transform these terms into epidemiological parameters with their statistical significances, these data may be reorganized as described in table 3.

**Table 3:** Re-arrangement of results from Table 2 for calculating OR or RR.

	Responders without ADR	Other results	Total
Drug A	a_1_	b_1_+c_1_+d_1_	n_1_
Drug B	a_2_	b_2_+c_2_+d_2_	n_2_

Focusing on the data contained in Table 3, let b_1_+c_1_+d_1_ = S_1_ and b_2_+c_2_+d_2_ = S_2_. Then a_1_*S_2_/a_2_*S_1_ expresses the odds ratio for “net efficacy adjusted for risk” (OR NEAR); and (a_1_/n_1_)/(a_2_/n_2_) expresses the relative risk for “net efficacy adjusted for risk” (RR NEAR). Finally, the 95% confidence intervals (CIs) for these new parameters may be calculated in the following manner [5]:
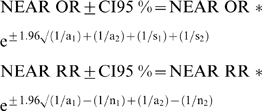



These NEAR OR and NEAR RR measures represent how much more beneficial and safe a proband drug is than the control drug and with what statistical significance. Moreover, these variables and their confidence intervals may be represented on a logarithmic scale, so that the statistical significance of differences between treatments may be visually determined. Naturally, the higher the sample size, the higher the accuracy of the measure.

To facilitate NEAR calculation, an Excel worksheet has been developed by the authors. Thus, numerical results as well as graphic display of NEAR OR and NEAR RR with their 95% CIs are easily obtained by running the application offered at the following website: http://investigacion-huc.com.

### A preliminary example of calculation and interpretation of results

To explain how to proceed in practice let us introduce a preliminary example. Efficacy and safety of two quinolones, gemifloxacin and trovafloxacin, were compared in a CRT for the treatment of community-acquired pneumonia [6]. Eligible patients were randomized to receive either oral gemifloxacin 320 mg once daily or oral trovafloxacin 200 mg once daily for seven days. The therapeutic response was defined as the combined clinical (no further antibiotic therapy was required) and bacteriological (original pathogens were erradicated) responses. Safety was assessed by recording adverse events with suspected or probable relationship to the study medication; treatment-associated adverse events leading to withdrawal were also recorded. As to explaining the procedure, we extracted results (by intention to treat analysis) corresponding to clinical response for benefit assessment and adverse events leading to withdrawal for safety assessment. Thus, 290 patients received gemifloxacin treatment; 254 of them (87.6%) exhibited a favourable clinical response at follow-up; 281 were treated with trovafloxacin, 228 of them (81.1%) responding favourably; 5.5% of patients (n = 16) in the gemifloxacin group and 4.6% (n = 13) in the trovafloxacin group had to be withdrawn due to treatment associated adverse events. The authors concluded that “gemifloxacin is an effective and well-tolerated treatment for patients with community-acquired pneumonia”.

Using the data obtained with gemifloxacin, we constructed Table 4 where the expected frequencies for each cell were easily calculated. After repeating calculations for trovafloxacin, Table 5 was constructed.

**Table 4:** Calculation of expected frequencies combining efficacy and safety with data belonging to a CRT in which gemifloxacin was assayed [6]. * = by rounding.

	Responders to gemifloxacin	Non-responders to gemifloxacin	
Not withdrawn	a = 254×274/290 = 240*	b = 36×274/290 = 34*	Total not withdrawn = 274
Withdrawn	c = 254×16/290 = 14*	d = 36×16/290 = 2*	Total withdrawn = 16
	Total responders = 254	Total non-responders = 36	Total studied = 290

**Table 5:** Expected frequencies combining efficacy and safety distributed according to the template shown in Table 3.

	Responders not withdrawn	Remaining patients	Total
Gemifloxacin	240	50	290
Trovafloxacin	217	64	281

Data are from a CRT in which gemifloxacin and trovafloxacin were assayed [6].

Now the odds ratio (NEAR OR) and the relative risk (NEAR RR) with their 95% confidence intervals may be easily computed. Table 6 shows the results obtained for these parameteres together with traditional odds ratio and relative risk for efficacy (i.e. without combining safety data) and for safety (i.e. without combining efficacy data). Classical NNT (number needed to treat) and NNH (number needed to harm) are also presented. In this manner the contribution of NEAR OR or RR to clinical trials or observational studies assesment may be compared with those of traditional measures. When the lower limit of the 95% confidence interval is above 1, the proband drug is deemed better than the control; in this case, gemifloxacin is the proband drug.

**Table 6:** Results of calculating NEAR and traditional efficacy and safety variables with data obtained from a CRT in which gemifloxacin was assayed versus trovafloxacin [6].

		Lower limit 95% confidence interval	Upper limit 95% confidence interval
Efficacy OR	1.64	1.04	2.60
Safety OR	0.83	0.39	1.76
**NEAR OR**	**1.40**	**0.93**	**2.12**
Efficacy RR	1.08	1.01	1.16
Safety RR	0.99	0.95	1.03
**NEAR RR**	**1.07**	**0.98**	**1.16**
NNT	−16	−8	−198
NNH	−101	−22	38

A lower limit of 95% confidence interval above 1.0 (except for NNT and NNH) means that gemifloxacin is preferable to trovafloxacin. The minus sign in the case of NNT indicates that fewer patients need to be treated with gemifloxacin to obtain one further success; in the case of NNH, the minus sign means that fewer patients need to be treated with gemifloxacin to produce one further adverse event.

On examining the data contained in the table, if only efficacy parameters are considered, gemifloxacin seems to be better than trovafloxacin; if only safety parameters are taken into account, no differences are found between treatments. Adjusting efficacy for safety in one measure, i.e NEAR OR and RR, it can be seen that no significant differences between the two treatments were observed. Therefore, the authors' narrative conclusion indicating that “gemifloxacin is an effective and well-tolerated treatment” may be better expressed by saying that according to the data reported gemifloxacin was as effective and safe as trovafloxacin.

### Assaying the model

To assay our model, a further series of examples selected from the literature was tested. Selection was made by applying the following criteria: firstly, cases arising from clinical practice, in which patient features required a particular management; in a more general setting, a second criterion was drugs recently introduced on the market, about which physicians usually like to know their actual advantages; and thirdly, cases in which prevention rather than a clinical change was the endpoint, safety being a critical aspect in these cases.

Finally, expected frequencies were compared with real frequencies. In this respect one rare study reporting the data required was used [1].

## Results

**Figure 1:**
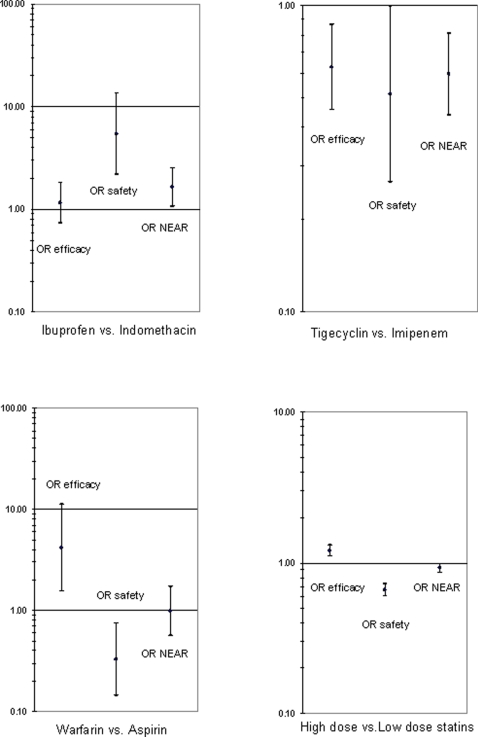
Graphic representation of traditional efficacy and safety odds ratio (OR) together with the OR for “net efficacy adjusted for adverse reactions” (NEAR ). Data were extracted from trials comparing ibuprofen vs. indomethacin for the closure of patent ductus arteriosus [7]; tigecycline versus imipenem/cilastatin in patients with complicated intra-abdominal infections [11]; warfarin vs. aspirin for preventing thromboembolic events in patients suffering atrial fibrillation [12] and more intensive vs. less intensive statin therapy in cardiovascular diseases [13]. When the lower limit of the 95% confidence interval is above 1.0, the proband drug (first-mentioned in graphic footnotes) is better than the control one.

In Figure 1, graphics for OR may be seen. This figure is an example of the possibilities of graphic representation of data. Numerical data for RR as well as for NNT and NNH are summarized in Tables 7, 8 and 9.

**Table 7:** Results of classical RR, NNT and NNH variables together with NEAR RR for a CRT comparing ibuprofen vs. indomethacin for the closure of patent ductus arteriosus [7].

	Ibuprofen vs. indomethacin		
		95% Confidence intervals	
Efficacy RR	1.04	0.92	1.18
Safety RR	1.14	1.07	1.22
**NEAR RR**	**1.19**	**1.02**	**1.38**
NNT	34	−8	16
NNH	7	11	5

Graphic representation of NEAR OR may be seen in Figure 1.

**Table 8:** Results of classical RR, NNT and NNH variables together with the new NEAR RR for a CRT comparing tigecycline versus imipenem/cilastatin in patients with complicated intra-abdominal infections [11].

	Tigecyclin vs. imipenem		
		95% Confidence intervals	
Efficacy RR	0.89	0.82	0.96
Safety RR	0.97	0.94	1.00
**NEAR RR**	**0.86**	**0.78**	**1.78**
NNT	11	35	7
NNH	−29	−15	−229

Graphic representation of NEAR OR may be seen in Figure 1.

**Table 9:** Results of classical RR, NNT and NNH variables together with the new NEAR RR for two CRT comparing. warfarin vs. aspirin for preventing thromboembolic events in patients suffering atrial fibrillation [12] and more intensive vs. less intensive statin therapy in cardiovascular diseases [13].

	Warfarin vs. aspirin			High vs. low dose statins		
		95% Confidence intervals			95% Confidence intervals	
Efficacy RR	1.05	1.02	1.08	1.02	1.01	1.03
Safety RR	0.95	0.92	0.99	0.97	0.96	0.98
**NEAR RR**	**1.00**	**0.96**	**1.05**	**0.99**	**0.98**	**1.00**
NNT	−22	−14	−62	−71	−49	−128
NNH	−20	−12	−56	−35	−29	−43

Graphic representation of OR may be seen in Figure 1.

### Cases arising from clinical practice

The first example corresponded to a comparison of ibuprofen and indomethacin for the closure of patent ductus arteriosus. The main concern for the pediatrician soliciting guidance was the occurrence of oliguria as an adverse effect. In this case, we retrieved a meta-analysis including eleven CRT in which both agents were compared [7]. From the eleven CRT, oliguria was recorded in three of them [8]–[10]. Working with pooled data, it may be seen that 191 infants received ibuprofen, ductal closure occurring in 140 of them (73.3%). Another 179 were given indomethacin and ductal closure occurred in 126 (70.4%). Oliguria occurred in 6 infants treated with ibuprofen and in 27 treated with indomethacin. The authors concluded that “ibuprofen was as effective as indomethacin; likewise, in general, there were no complications with the treatments, except for a risk reduction in suffering oliguria with ibuprofen”. However, this risk reduction in suffering oliguria was not statistically analyzed by the authors. In this manner, OR and RR for efficacy and NNT revealed that both compounds had a similar efficacy; OR and RR for safety, and NNH, indicated that ibuprofen is safer than indomethacin. Expected frequencies calculation indicated that ductal closure was obtained without suffering oliguria in 136 out of 191 patients (71.2%) in the ibuprofen arm and in 107 out of 179 in the indomethacin arm (59.7%). Both NEAR OR and RR confidence intervals confirmed that ibuprofen was significantly better than indomethacin (Figure 1 and Table 7). Therefore, in this case, our model helped to discriminate ibuprofen as a statistically significant better option.

### Examples with drugs recently introduced on the market

In the second setting, an appraisal of a new drug was performed.

Tigecycline is a novel antibiotic with in vitro activity against microorganisms frequently associated to intra-abdominal infections; on the other hand, in hospitalized patients with complicated infections, imipenem/cilastatin is the drug of choice. A multicenter CRT studied the efficacy and safety of tigecycline as compared with imipenem/cilastatin in complicated hospitalized intra-abdominal infections [11]. Four hundred and eight clinically evaluable patients received tigecyclin and 399 imipenem/cilastatin. At the test-of-cure visit, 69.7% patients of the tigecyclin group and 78.4% of the imipenem/cilastatin group were clinically cured. When withdrawals from the study caused by drug problems were computed, 6.5% in the tigecycline arm and 3.6% in the imipenem/cilastatin arm were noted. The authors concluded that “tigecycline is an effective and well tolerated monotherapy option for the treament of patients with complicated intra-abdominal infections, with comparable efficacy to imipenem/cilastatin”. However, traditional OR and RR for efficacy and NNT indicate that the control group was better than proband; no differences were found for safety, except for NNH which was clearly favourable to the control drug. Expected frequencies calculation indicate that 266 out of 408 (65.2%) in the tigecyclin arm and 302 out of 399 (75.7%) in the imipenem/cilastatin one improved without suffering adverse reactions. Results of our model (Figure 1 and Table 8) clearly indicated that imipenem/cilastatin, the classical drug of choice, continued to be a better option than tigecycline in treating this type of infection.

### Cases in which disease prevention is the endpoint

A third setting is related to those studies in which preventing a harmful event is the primary endpoint. Most of these studies correspond to secondary prevention of events arising in the course of chronic diseases, needing therefore long lasting treatments. In these cases, there is no doubt that safety is a critical aspect. Given the relevance of this setting, two examples will be shown.

A first example of this circumstance comes from a generalized debate among cardiologists as to the use of warfarin versus aspirin to prevent thromboembolic complications in chronic atrial fibrillation. In this case a randomized 2-year comparative trial of warfarin, aspirin and placebo was retrieved [12]. In calculating NEAR, the data corresponding to placebo were not included. The aspirin dose was 75 mg/day, and that of warfarin was adjusted by the international normalized ratio. Of 335 patients treated with warfarin, 330 did not suffer thromboembolic complications (98.5%); of 336 patients treated with aspirin, 316 did not suffer thromboembolic complications (94.0%). The authors concluded that it was recommendable to use warfarin to prevent thromboembolic complications, which today constitutes routine practice in these patients. We found that traditional OR and RR for warfarin efficacy as well as NNT significantly favored this compound; the opposite was the case with the safety variables, all of them favoring aspirin when severe gastrointestinal and pulmonary hemorrhage were considered (23 versus eight in proband and control groups, respectively). By calculating the expected frequencies, 307 out of 335 patients (91.6%) in the proband group and 308 out of 336 in the control one (91.6%) were protected without suffering from severe hemorrhage. When both NEAR OR and RR were calculated, the difference between treatments disappeared (Figure 1 and Table 8). In view of these results, one wonders whether the clinical benefit obtained is really so important as to continue recommending warfarin for this kind of patients.

Another example of disease prevention comes from the more intensive versus less intensive therapy with statins for secondary reduction of cardiovascular events and stroke. A recently published meta-analysis deals with this subject [13], in which results of seven trials were pooled; pravastatin, simvastatin, atorvastatin and lovastatin were the drugs studied; 13,641 of 14,768 patients (92.4%) receiving high dose therapy were protected from myocardial events whereas 13,302 of 14,625 (91.0%) receiving low dose therapy were also protected. According to the authors, a non-significant difference of 7.8% v.s. 5.3% of patients (high versus low dose, respectively) discontinued drug therapy as a consequence of an adverse event. The authors concluded that “in summary, more intensive statin therapy is safe and well-tolerated. It provides incremental benefits over and above those of lower intensity statin therapy in the secondary prevention of myocardial infarction and stroke in patients with known coronary disease”. Indeed, classical parameters confirm the authors' conclusion as to efficacy; however, as to safety, all the variables indicate that low dose is preferable. Expected frequencies calculation showed that with the high dose regime statins protected 12,577 out of 14,768 patients (85.2%) without causing ADR; this value was 12,597 out of 14,625 (86.1%) in the case of the low dose regime. By adjusting efficacy to risk in one measure, it is clearly seen that no significant differences exist between the two treatment schedules (Figure 1 and Table 8). Therefore, since muscle disturbances are a well known adverse effect of these drugs, a more conservative recommendation as to using high dose may be more suitable.

### A comparison of expected vs. real frequencies

Finally, the accuracy of expected frequencies was tested against frequency data corresponding to a CRT reported in the literature [1]. This is one of the rare cases in which the authors detailed the actual values of patients improved without suffering ADR; the authors compared an unknown test drug with a standard one jointly with hydrochlorothiazide to control blood pressure. With these data we calculated the expected frequencies for each group, comparing them with real frequencies. The results are summarized in Table 10 (excluding drop-outs). As can be seen, expected frequencies suitably fit actual values.

**Table 10:** Comparison of real and expected frequencies with data extracted from a clinical trial in which hydrochlorothiazide plus a test drug (T) was compared with hydrochlorothiazide plus a standard drug (S) in hypertensive patients [1].

	Responders without ADR		Responders suffering ADR		Non-responders non suffering ADR		Non-responders suffering ADR	
	Actual value	Expected frequency	Actual value	Expected frequency	Actual value	Expected frequency	Actual value	Expected frequency
HCTZ+T	50	54	153	149	14	10	24	28
HCTZ+S	101	108	40	33	82	75	16	23

## Discussion

NEAR OR and RR calculation represent a simple and useful tool for combining risks and benefits in one easily understandable measure. Moreover, data for calculation may be easily obtained from published CRT. Lastly, NEAR may help in solving clinical problems arising in practical settings. Therefore, the objectives outlined in the Introduction have all been accomplished. Nevertheless, certain topics deserve discussion.

First, although NEAR is a similar approach to a binary composite endpoint defining “efficacy without ADR”, it is not exactly equivalent. Using a binary endpoint with such components requires knowing the number of patients that improved without being affected by ADR, which is the obstacle we have tried to overcome with our method. Recently, a method for analyzing a binary composite endpoint when there are missing data in components has been reported [14], but such a mathematically complex analysis is only viable when the CRT has been designed with the binary endpoint “efficacy without ADR” as the main variable. Our approach lends support to this kind of analysis and emphasizes the necessity of designing clinical trials with these composite endpoints in which efficacy is balanced against safety.

Second, there is no doubt that mathematical formulations developed by other authors are highly interesting. For instance, the use of risk:benefit contours in cancer therapy [2] may represent a relevant contribution. Similarly, the conversion of traditional NNT into NNT_us_ (NNT unqualified success) and NNT_uf_ (NNT unmitigated failure) [3], which allow us to combine efficacy and safety, is also an attractive approach. Perhaps other proposals, although evidently relevant from a theoretical point of view [1], [4], may be excessively complex for translation to clinical practice. Anyhow, it is clear that none of these formulations have attained the popularity of NNT and NNH [15]; the likely reason for this is that these parameters represent an easily understandable and simple concept, i.e. number of patients needed to treat to obtain one further success or one additional adverse event, respectively. Theoretically, an NNT/NNH ratio could be a useful measure combining efficacy and safety, but calculating these parameters may produce numbers with negative or positive values, which makes it difficult to interpret a ratio obtained by dividing them. In addition, when their confidence intervals include negative and positive values the possibility of obtaining rational results is practically null. For this reason, we opted for a procedure in which arithmetical signs were avoided.

Third, an important item in our model is the interpretation of results. In this respect, these new measures must be interpreted rigorously within the context of the problem features, caution being exercised in extrapolating conclusions beyond this frontier. In other words, usefulness of this new model depends on suitably selecting those efficacy (benefit) and safety (risk) variables that best define the problem. The varying importance of these variables depends on the case under consideration. For instance, the risk of pruritus produced by an anticancer treatment is less important than the benefit; whereas it becomes more important in cases only needing antiacid therapy. In this respect, efficacy variables are thoroughly reported in most published reports of CRT, and selecting one of them is extremely easy, whereas adverse reaction descriptions are too often meager and confusing. Therefore, in selecting a safety variable, the concrete clinical problem intended to treat should be considered (v.g. oliguria and NSAID). In the case of more general analyses, an appropriate variable for safety may be the proportion of patients that discontinued drug treatment as a consequence of an ADR (the case of high v.s. low dose statins).

Continuing with result interpretation, the 95% CIs used in our procedure are widely used in OR and RR calculation, and, consequently, the assumptions under which these CIs are correct are the same for NEAR OR and NEAR RR. In this respect, although the CIs were calculated with “transformed” (expected) values, theoretically these “transformed 95% CIs” must presumably have a similar level of certainty to that obtained by using real (observed) values. In fact, on calculating CIs with the data shown in Table 10, in which observed and expected values obtained in a real clinical trial [1] are shown, the 95% CIs for OR and RR for observed values were practically identical to those calculated with expected values. Indeed, in the case of observed values, the NEAR OR was 0.357 (0.238–0.535) and in the case of expected values the result was 0.350 (0.235–0.520). For RR the results were similar. Therefore assumptions applied to “observed 95% CIs” may be similarly applied to “transformed 95% CIs”. However, in practice, when the upper or the lower limit of NEAR OR or NEAR RR 95% CIs are quite close to 1.0, say 0.95 or 1.05, a wise use of the measure should require “a post hoc” discussion and perhaps a revision of complementary information; a similar situation may arise when considering wide CIs and/or small sample sizes. Anyhow, these are not specific problems of NEAR interpretation; these are general problems of working with CIs.

Fourth, according to evidence-based medicine, the quality of clinical trials analyzed is a critical factor for obtaining reliable conclusions. In fact, only high quality CRT or high-quality CRT-based meta-analyses should be employed. In this respect, a simple five-score scale to measure CRT quality has gained wide acceptance [16]. Although Tables 7, 8 and 9 do not show these scores (to avoid cluttering), it can be said that the data used were of high quality.

Fifth, together with quality, the CRT employed must satisfy other technical requirements. So, only those trials describing efficacy and safety as categorical variables may be analyzed by our procedure. In contrast, those studies in which efficacy and safety are not measured in the same series of patients cannot be used. Special care must be taken with ADR data, since a single patient may often present several adverse symptoms, so the number of ADR may be higher than the number of patients, which can produce errors in calculation. Finally, a limitation of working with categorical variables is that the degree of improvement or deterioration balance may only be determined when the authors report such detail. Indeed, when the authors indicate that the endpoint is, say, an increase in the magnitude of parameter x, we can determine the degree of improvement; but if they only indicate that the endpoint is, say, any decrease of temperature, the magnitude of the antipyretic effect cannot be determined.

Sixth, when the results obtained with traditional tools for measuring efficacy or safety separately were compared with NEAR OR or NEAR RR, it is clear that these new tools tend to yield balanced results. In this manner, we can have a global and mathematical appraisal of two therapeutic options by means of a single view.

Finally, we leave open the possibility of using NEAR OR or NEAR RR. In this respect, there is a debate as to which parameter, OR or RR, should be used [17]. Apparently, some authors think that preferring one to another is a matter of patient setting as well as the type of design used in the study, i.e. experimental versus observational. Therefore, we have presented the two parameters to leave open the possibility of using one over the other.

In conclusion, NEAR representing overall risk:benefit as measured in the present study may contribute to improving knowledge of drug usefulness and facilitate the rational choice of drugs by prescribers. Within these general comments it must be emphasized that narrative results without accurate data about particular patients affected by ADR are often the only source of information, which radically contrasts with the almost exquisite and detailed manner in which efficacy is reported. In this respect, our measure represents an effort to change this way of thinking.

## References

[1] Chuang-Stein C, Mohberg N, Sinkula M (1991). Three measures for simultaneously evaluating benefit and risks using categorical data from clinical trials.. Stat Med.

[2] Schulzer M, Mancini G (1996). ‘Unqualified success’ and ‘unmitigated failure’: number-needed-to-treat-related concepts for assessing treatment efficacy in the presence of treatment-induced adverse events.. Int J Epidemiol.

[3] Shakespeare T, Gebski V, Veness M, Simes J (2001). Improving interpretation of clinical studies by use of confidence levels, clinical significance curves, and risk:benefit contours.. Lancet.

[4] Tubert-Bitter P, Bloch DA, Raynauld (1995). Comparing the bivariate effects of toxicity and efficacy of treatments.. Stat Med.

[5] Hartzema A, Koch G, Hartzema A, Porta M, Tilson H (1991). Basic statistical methods in pharmacoepidemiologic study designs.. Pharmacoepidemiology.

[6] File T, Schlemmer B, Garau J, Cupo M, Young C, and the 049 Clinical Study Group (2001). Efficacy and safety of gemifloxacin in the treatment of community-acquired penumonia: a randomized, double-blind comparison with trovafloxacin.. JAC.

[7] Gimeno A, Modesto V, Morcillo F, Fernández C, Izquierdo I, Gutiérrez A (2007). Ibuprofen versus indomethacin in the preterm persistent patent ductus arteriosus therapy: review and meta-analysis.. An Pediatr.

[8] Gimeno A, Cano A, Fernández C, Carrasco J, Izquierdo I, Gutiérrez A (2005). Ibuprofeno frente a indometacina en el tratamiento del conducto arterioso persistente del prematuro.. An Pediatr.

[9] Lago P, Bettiol T, Salvadori S, Pitassi I, Vianello A, Chiandetti L (2002). Safety and efficacy of ibuprofen versus indomethacin in preterm infants treated for patent ductus arteriosus: a randomised controlled trial.. Eur J Pediatr.

[10] Van Overmeire B, Smets K, Lecoutere D, Van de Broek H, Weyler J, Degroote K (2000). A comparison of ibuprofen and indomethacin for closure of patent ductus arteriosus.. N Engl J Med.

[11] Oliva M, Rekha A, Yellin A, Pasternak J, Campos M, Rose G (2005). A multicenter trial of the efficacy and safety of tigecycline versus imipenem/cilastatin in patients with complicated intra-abdominal infections.. BMC Infect Dis.

[12] Petersen P, Boysen G, Godtfredsen J, Andersen E, Andersen B (1989). Placebo-controlled, randomised trial of warfarin and aspirin for prevention of thromboembolic complications in chronic atrial fibrillation.. Lancet.

[13] Josan K, Majumdar S, McAlister F (2008). The efficacy and safety of intensive statin therapy: a meta-analysis of randomized trials.. CMAJ.

[14] Quan H, Zhang D, Zhang J, Devlamynk L (2007). Analysis of a binary composite endpoint with missing data in components.. Statist Med.

[15] Laupacis A, Sackett DL, Roberts RS (1988). An assessment of clinically useful measures of the consequences of treatment.. N Engl J Med.

[16] Jadad AR, Moore RA, Carroll D, Jenkinson C, Reynolds DJ, Gavaghan DJ (1996). Assessing the quality of reports of randomized clinical trials: is blinding necessary?.. Control Clin Trials.

[17] Schechtman E (2002). OR, RR, absolute risk reduction, and the number needed to treat. Which of these should we use?.. Value Health.

