# Correction: Net Efficacy Adjusted for Risk (NEAR): A Simple Procedure for Measuring Risk:Benefit Balance

**DOI:** 10.1371/annotation/25fa551c-b31c-438b-827f-8b6caa83a65c

**Published:** 2010-08-06

**Authors:** José N. Boada, Carlos Boada, Mar García-Sáiz, Marcelino García, Eduardo Fernández, Eugenio Gómez

The Excel worksheet used to facilitate NEAR calculation has been replaced by an informatic application. Numerical results as well as graphic display of NEAR OR and NEAR RR with their 95% CIs are easily obtained by downloading the freely available application offered at the following Web site: http://www.fitec.ull.es/beneficioriesgo_en.html

